# Toll-Like Receptors (TLRs): Structure, Functions, Signaling, and Role of Their Polymorphisms in Colorectal Cancer Susceptibility

**DOI:** 10.1155/2021/1157023

**Published:** 2021-09-12

**Authors:** Aga Syed Sameer, Saniya Nissar

**Affiliations:** ^1^Basic Medical Sciences Department, College of Medicine, King Saud bin Abdulaziz University for Health Sciences, King Abdullah International Medical Research Centre (KAIMRC), National Guard Health Affairs (NGHA), King Abdulaziz Medical City, Jeddah 21423, Saudi Arabia; ^2^Molecular Diseases & Diagnostics Division, Infinity Biochemistry Pvt. Ltd, Sajjad Abad, Chattabal, Srinagar, Kashmir 190010, India; ^3^Department of Biochemistry, Government Medical College, Shri Maharaja Hari Singh Hospital, Karan Nagar, Srinagar, Kashmir 190010, India

## Abstract

Toll-like receptors (TLRs) are the important mediators of inflammatory pathways in the gut which play a major role in mediating the immune responses towards a wide variety of pathogen-derived ligands and link adaptive immunity with the innate immunity. Numerous studies in different populations across the continents have reported on the significant roles of TLR gene polymorphisms in modulating the risk of colorectal cancer (CRC). CRC is one of the major malignancies affecting the worldwide population and is currently ranking the third most common cancer in the world. In this review, we have attempted to discuss the structure, functions, and signaling of TLRs in comprehensive detail together with the role played by various TLR gene SNPs in CRC susceptibility.

## 1. Introduction

Colorectal cancer (CRC) is one of the commonly diagnosed cancers representing about 10% of all cancers worldwide and is currently ranked as the third most common cancer in men and second most in women worldwide [1–3]. CRC usually arises from the inner wall of the intestines and does involve the colon rectum and appendix [4].

CRC is among few solid tumors in which inflammation acts as a founder effect and plays a key role in the carcinogenesis mechanism in transforming the normal cells into the malignant ones [5, 6]. In addition, numerous researchers have demonstrated a positive association between various innate immune system mediators and bacterial toxins with the development of CRC have also been found [7–9]).

CRC has a multifarious etiology and is usually classified as sporadic, inherited, or familial in their origin and occurrence [10]. With respect to the risk factors, age, sex, and race, the inflammatory diseases are recognized as the prime ones for the development of the CRC [11]. CRC initiation and progression follow a multigene and multistage genetic instability model called the Vogelstein model of carcinogenesis [12]. Numerous gene mutations (suppressing and activating), gene silencing, and genetic polymorphisms have been shown to play their role in driving the carcinogenesis pathway, with each stage having a unique molecular fingerprint of genetic variations [10, 11].

Currently, three pathways for the genetic instability have been identified for the CRC initiation, development, and progression which are as follows: chromosomal instability (CIN) pathway, microsatellite instability (MSI) pathway, and CpG island methylator phenotype (CIMP) pathway [11, 13, 14].

Toll-like receptors (TLRs) have been identified as the important mediators of inflammatory pathways in the gut which serve as crucial regulators in maintaining the balance between commensal bacteria in the gut and the mucosal immune system [15–20]. Numerous studies across the populations and across the continents have reported on the significant roles of *TLR* gene polymorphisms modulating the risk of CRC [21–28].

In this review, we aim to sketch an in-depth picture about the importance of TLR functions, their signaling, and the role genetic polymorphisms of some of the important and common *TLR* genes do play in the colorectal cancer susceptibility.

## 2. Toll-Like Receptors (TLRs)

Toll-like receptors (TLRs) are evolutionarily conserved receptors belonging to the family of pattern recognition receptors (PRRs) which play a vital role in immune responses especially pathogen recognition by the extracellular matrix [18, 29–31]. TLRs are directly involved in the regulation of inflammatory reactions and activation of the innate or adaptive immune responses for the elimination of infectious pathogens and cancer debris [29, 32–34].

TLRs hold a key position in the first line of defense against pathogens because of their ability to recognize the conserved pathogen-associated molecular patterns (PAMPs), conserved structures of the pathogens, or the damage caused by the pathogens within the host [35–38].

Pattern recognition receptors (PRRs) make up a key component of the innate immunity because of their ability to initially sense the exposure to infection and elicit an intracellular signaling cascade for the eventual elimination of the pathogen and infected cell thereof [18]. All PRRs are germline-encoded proteins which can recognize wide varieties of alien molecules (e.g., lipid, carbohydrate, peptide, and nucleic acid) commonly found in pathogens but distinct from the host molecules and thus are commonly referred to as PAMPs [39]. Activation of PRRs results in the downstream transcriptional activation and expression of numerous inflammatory mediators [18, 35, 36, 38]. In addition, PRR signaling also leads to the triggering of various processes involved in autophagy, cell death, cytokine processing, and phagocytosis [40, 41].

Currently, based on their composition and structure, PRRs are categorized into five classes depending on their primary functions, position, specificity for ligands, and evolutionary associations as follows:

- Toll-like receptors (TLRs)
- AIM2-like receptors (ALRs)
- C-type lectin receptors (CLRs)
- Retinoic acid-inducible gene- (RIG-) I-like receptors (RLRs)
- NOD-like receptors (NLRs) [18, 35]

TLRs and CLRs function as membrane-bound receptors and exist as transmembrane proteins, while others occur as cytosolic proteins [18, 35, 42]).

PRRs have been found to be expressed not only in specialized immune response cells like macrophages, neutrophils, dendritic cells (DKs), natural killer (NK) cells, mast cells, basophils, and eosinophils but also on other cells as well [32, 35].

Among the numerous molecular patterns which PRRs can recognize as foreign and be able to elicit an innate immune response, four are characterized till date which are as follows: pathogen-associated molecular patterns (PAMPs), damage/danger-associated molecular patterns (DAMPs), microbe-associated molecular patterns (MAMPs), and xenobiotic-associated molecular patterns (XAMPs). Most of the PRRs after getting activated by the molecular patterns from the pathogens cause the upregulation of the inflammatory response gene especially proinflammatory cytokines and type I interferons [18, 35, 42]. While TLRs and CLRs are sensitive to the molecular patterns located outside the cell because of their surface expression, others respond to the pathogen-derived patterns intracellularly.

### 2.1. TLRs: History, Structure, and Functions

The discovery of the TLR receptors dates to the identification and functional characterization of the toll gene and its protein in Drosophila [43]. TLRs received their name because of their similarity to the toll protein in serving as the cell surface receptors [44], which play a role in providing the immunity against the fungal and gram-positive bacterial infections [45] together with their role in embryonic development and dorsoventral polarization [35, 46–49]).

**Figure 1:**
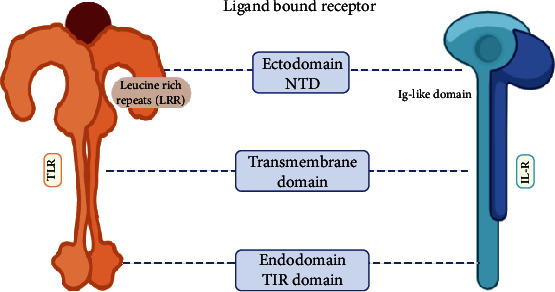
A comparative structure of TLR and IL-R.

TLRs are categorized as a type of type I integral transmembrane proteins, usually consisting of three domains—an N-terminal domain (NTD) located outside the membrane, a middle single helix transmembrane domain traversing the membrane, and a C-terminal domain (CTD) located towards the cytoplasm [50]. N-terminal domain makes up an ectodomain to serve as the ligand recognition site to the variety of PAMP, while CTD is involved in the interactions with various signal transduction adaptors for the initiation of downstream signaling through its toll-IL-1 receptor (TIR) homologous domain [18, 35, 42, 48, 50]. The ectodomain has a folded solenoid structure (resembling a horseshoe) of about 19-25 highly conserved short tandem leucine-rich repeat (LRR) motifs, which provide the much-needed specificity and recognizability to the TLRs for PAMPs/DAMPs [36, 51]. Each LRR repeat sequence of the TLRs contains 24-29 amino acids in a sequence pattern of xLxxLxLxx [35, 52]. In addition, the NTD does contain glycan moieties which serve as the actual binding sites for various pathogen-derived ligands [29, 53].  Figure 1 provides a comparison between the two receptors.

### 2.2. TLR Family Members

TLRs are further subdivided into two groups, depending upon the number of cysteine clusters present within the extracellular LRR motif as follows: vertebrate type (V-Type) and protostome type (P-Type). V-Type TLRs possess a single cluster of cysteine or CF motif on their CTDs (LRRCT) and hence also referred to as Single Cysteine Cluster TLRs (sccTLRs), and P-Type TLRs have multiple (two or more) cysteine clusters or CF motifs either on their LRRCT or on the N-terminal (LRRNT) domain [54, 55].

The TLR family plays a critical role in the immune system of an individual, be it an insect or a human being. They are one of the best-characterized PRR families, which function to recognize the self and nonself antigens, detect various pathogens, bridge the innate and adaptive immunity, regulate cytokine production, and regulate proliferation and survival of the host cell [18, 35, 48, 56].

To date, 222 TLRs have been identified in invertebrates and 28 TLRs in vertebrates, with the highest number of TLRs in an individual found in the vertebrate fish of teleost which possesses 21 TLR molecules—TLR1-5, TLR7-9, TLR13-14, TLR18-23, and TLR25-28 [35, 42, 48, 55, 57, 58] (Table 1). In mammals, a total of 13 TLRs (TLR1-13) have been found—however, humans possess only 10 (TLR1-10) of them while mouse possesses all with TLR10 being nonfunctional [35, 42, 48, 57]. Much of the ligands and the signaling pathways of the TLR1-9 and 11 are known, while the biological role of TLR10, 12, and 13 still is not clear. In humans, TLR4 was the first of the TLR family to be discovered and reported to regulate the inflammatory responses [59], while TLR3 is the most ancient one which belongs to the viral TLRs like TLR7-9 [35, 53].

Depending upon their functionality and location in the host cell, TLRs are further categorized into two types [18, 35, 38, 48, 56] as follows:

1. *Cell membrane TLRs*, which are expressed in their active form on the cellular surface, with NDT ectodomain facing outside for sensing and binding with the alien ligands. They include TLR1, 2, 4, 5, 6, and 10. In their active signal-transducing form, they exist as dimers—TLR1-TLR2, TLR2-TLR6, and TLR2-TLR4/5/10 [60]
2. *Intracellular TLRs*, which are expressed within the host cells on the organelle biomembranes like endoplasmic reticulum (ER), endosomes, and lysosomes. They include TLR3, 7, 8, and 9 [52, 61]

## 3. TLR Ligands

**Figure 2:**
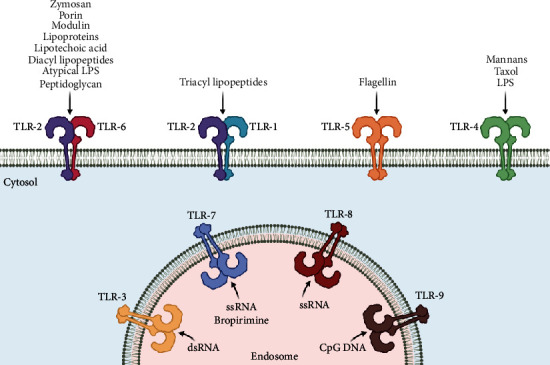
Detection of PAMPs by various TLRs.

TLRs are predominantly expressed in the host antigen-processing cells (APCs) which play crucial roles in recognizing the pathogenic infection and deciding their fate [62, 63]. TLRs have evolved to recognize a diverse array of PAMPs from a range of microorganisms including bacteria, protozoa, fungi, and viruses [20, 52, 53, 62, 63]. For almost each and every kind of major biomolecules derived exogenously from the pathogens (PAMPs) be it a protein, carbohydrate, lipid, or a nucleic acid, there exist one or more TLRs in the host cell for its binding and recognition as a foreign entity (Figure 2) [20, 52, 63]. Similarly, TLRs also do bind with various endogenous ligands (host-derived DAMPs) like plasma membrane constituents, heat shock proteins (HSPs), and organelle nucleic acids like mitochondrial DNA (mt DNA) [64].

However, TLR2 and TLR4 are the most efficient of the PRRs in being able to recognize and consequently get activated by a wide variety of ligands [20, 63] (Table 2). Some of the TLRs are highly specific in recognizing and binding to some particular ligands, like TLR2 for lipoproteins, lipotechoic acid (LTA), peptidoglycans (PG), zymosan and porin [65, 66], TLR3 for dsRNA derived from viruses [67], TLR4 for gram-negative bacteria-derived lipopolysaccharides (LPS) and plant-derived taxol [64, 68], TLR5 for the gram-negative bacterial flagellin protein [69], and TLR7 for antiviral synthetic compounds—imidazoquinolines and loxoribine [70, 71].

## 4. TLR Signaling Pathways

On activation by binding of the ligand to the LRR domain of TLR, the signal needs to be transferred to the internal environment of the cell. For TLRs, various adaptor molecules have been identified which get recruited to the CTD domain of the activated and/or dimerized receptor to pass the signal downstream of the cell [31, 72, 73]. The important adaptors which are critical for the functioning of the TLR signaling are identified by the possession of the toll-IL-1 receptor (TIR) domains in their structure and are categorized as follows:

- Myeloid differentiation primary-response protein 88 (MyDD88)
- TIR domain-containing adaptor protein (TIRAP or Mal)
- TIR domain-containing adaptor inducing IFN-*β* (TRIF, TICAM1)
- TRIF-related adaptor molecule (TRAM, TICAM2)
- B-cell adaptor for phosphoinositide (BCAP)
- Sterile *α*- and armadillo-motif-containing protein (SARM)

**Figure 3:**
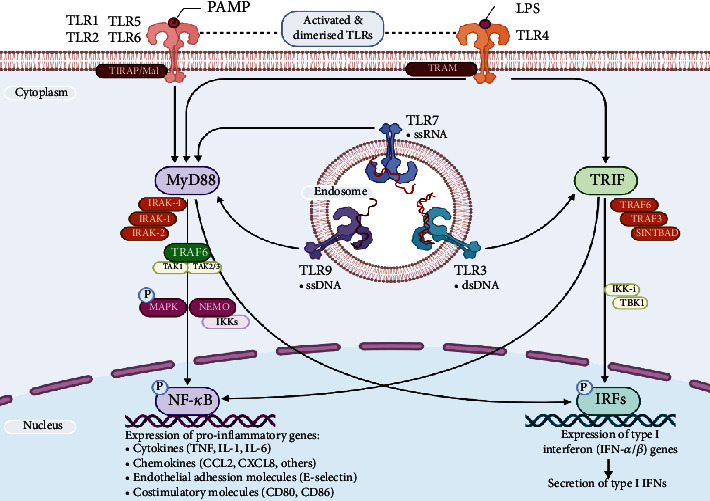
Dynamics of the toll-like receptor signaling.

Since TLRs belong to the class I transmembrane receptor family, the binding of the ligand induces a dimerization which in turn activates them [20, 31, 35, 52, 63]. The actual signaling cascade is dependent upon the type of ligand, interacting TLR, and the downstream adaptor molecule engaged for the signaling pathway. However, TLR signaling is generally divided into two distinct pathways based on the recruitment of either MyD88 or TRIF for the downstream signaling cascades [18, 31, 35].  Figure 3 sketches the essential dynamics of the two pathways of TLR signaling.

MyD88-dependent pathway

It is utilized by all TLRs (except TLR3) which eventually lead to the enhanced expression of proinflammatory cytokine genes. MyD88 recruitment to the activated CTD domain of the TLRs is facilitated by two important adaptor molecules—Mal and TIRAP. Mal adaptor protein is utilized by all kinds of TLR receptors to recruit MyD88, while TLR1, 2, 4, and 6 require additional association with TIRAP molecule to facilitate the contact with MyD88 [52].

TRIF-dependent pathway

It is utilized by TLR3 and TLR4 to trigger the enhanced expression of interferon type-1 genes. TLR3 utilizes it in response to the binding of viral dsRNA by recruiting TRIF directly, while TLR4 requires another adaptor protein, TRAM, for the activation of TRIF [18].

### 4.1. Effect on Carcinogenesis

**Figure 4:**
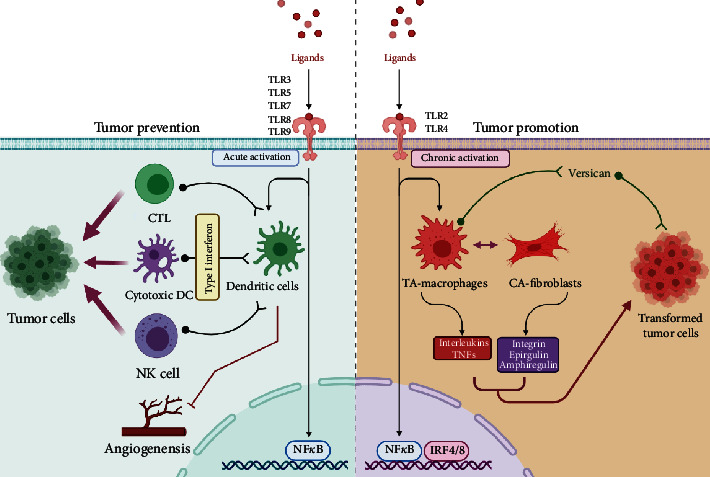
The two faces of the TLR signaling in carcinogenesis.

The specific ligand activation of the TLR signaling has dual and contrasting functions on the carcinogenesis pathways. On the one hand, TLR signaling has been shown to promote the carcinogenesis through proinflammatory, antiapoptotic, proliferative, and profibrogenic signals within the tumor microenvironment or tumor cells themselves; while on the other hand, TLR signaling induces a highly sensitive and effective tumor immunosurveillance by activating specific immune cells like dendritic cells (DCs), natural killer cells (NKs), and cytotoxic T lymphocytes (CTLs), all which have antitumorigenic activity [74].  Figure 4 summarizes the two faces of the TLR signaling in carcinogenesis.

Activation of one class of TLRs in particular TLR3, 5, 7, 8, and 9 does play a role in providing the antitumor immunity by modulating the function of immune tolerance via type I interferon [18, 31]. Primary modulation occurs via DC activation, which otherwise are chief mediators of immunosurveillance [75]. Within the tumor environment, DCs serve as the chief antigen-presenting cells (APCs) by expressing large number of PRRs including TLRs. DCs mediate the antigen processing and presentation for both innate and adaptive immune responses. On activation, DCs exert their effective modulating role on the key effectors like NKs and CTLs in a type I interferon-dependent manner [74, 76]. Additionally, TLR-induced expression of alpha IFNs is known to limit tumorigenesis by suppressing the angiogenesis [77, 78].

On the other hand, ligand-specific low and chronic stimulation of TLR2 and 4 leads to the tumor-promoting effects by enhancing inflammatory and antiapoptotic signals. The central role in both these effects is played by the key transcription factor NF-*κ*B [74]. NF-*κ*B has two-prong effects on the cellular environment—on the one hand, the NF-*κ*B-dependent TLR pathway enhances the overexpression of numerous proinflammatory molecules including interleukin- (IL-) 1b, tumor necrosis factor a (TNFa), and IL-6 [18, 31, 79–81]; while on the other hand, it leads to the increased expression of many antiapoptotic genes in addition to the restriction of proapoptotic pathways mediated by c-Jun kinase [82, 83]. Moreover, within the tumor microenvironment, two important TLR expressing cells, i.e., tumor-associated macrophages (TAMs) and cancer-associated fibroblasts (CAFs), have been reported to provide survival signals for tumor growth [74]. Also, within the tumor microenvironment, there is an increased expression and secretion of versican, a large ECM proteoglycan, which is an important component of tissue inflammation as a result of infection or injury [84]. Versican results in further enhanced activation of TLR signaling thereby amplifying and fueling the tumor-promoting inflammatory signals furthermore [74].

## 5. Toll-Like Receptor Polymorphism and Colorectal Cancer

TLRs are important cogwheels of the immune machinery. Because of their ability to recognize and bind with a wide variety of PAMPs (bacteria, parasites, fungi, and viruses) and DAMPs, they act as important regulators of the immune responses and bridge the innate with adaptive immune responses in the host [20, 31, 35, 52, 63, 85]. Majority of the immune response elicited by the TLRs is due to the highly specific ectodomain containing a germ line encoded and highly conserved LRR region [18, 31, 53]. Much of the ligand specificity of each TLR is because of this LRR region [18].

Various TLRs play a critical role in innate immunity by responding to the various PAMPs (from the pathogenic bacteria, viruses, or fungi) which drive the cellular response producing proinflammatory cytokines, chemokines, and other mediators, thus playing an important role in inflammatory reactions and activation of the adaptive immune pathways as well [86–88]. TLR-induced proinflammatory responses are considered as the first line of host defense, not only working against the pathogens but also accelerating the healing process to restore the immune homeostasis [89].

It has been demonstrated that any dysregulation in the TLR signaling, be it in ligand recognition and binding or in downstream signaling, has a founder effect in various immunopathological diseases like autoimmune diseases, allergy, arthritis, atherosclerosis, tuberculosis, rheumatic disorders, pancreatitis, and malignancies [22, 24, 88–93].

Any genetic variations (GVs) be it a mutation or a polymorphism which affects the *TLR* genes thereby affects the structure of TLRs especially their ligand-binding domains (NTD-LRR) and intracellular (CTD). TIR domain may play a role in modulating the incidence, the severity, and the outcome of many immune-related diseases [89, 94]. With the advent of technology and easy sequencing techniques, several studies have demonstrated the existence of numerous genetic polymorphisms affecting almost all *TLR* genes which have influence on the immune responses of the host towards the pathogenic infection or the consequent PAMPs and DAMPs [19, 22, 85, 89, 94].

In colorectal carcinogenesis, inflammation has been demonstrated as a major risk factor, with the involvement of different driver molecules like NF-*κ*B, interleukins, tumor suppressor proteins, oncogenes, cyclooxygenase, nitric oxide synthases, and growth factors including TLRs [85, 86, 95]. Numerous studies have linked the dysregulation of the TLR expression to the colorectal carcinogenesis [16, 26, 27, 96–100], and now, the evidence is mounting for the role of genetic polymorphisms in modulating the risk of CRC in various populations as well [17, 19, 21, 22, 24, 28, 49, 89, 94].

Of all the TLRs that are functional in humans, the single nucleotide polymorphisms (SNPs) of five *TLR* genes—*TLR2*, *3*, *4*, *5*, and *9*—have been reported to be involved in modulating the risk of CRC extensively [17, 21, 22, 25, 28, 49, 101, 102]. SNPs in any gene results in the change of amino acid sequence (missense/frameshift) which alters the structure and activity of the involved protein [103]. Numerous SNPs in the TLR gene especially in the NTD-LRR domain result in the disruption of the pathogen recognition ability as well as immune functions of these receptors (see Table 3) [89, 94].

### 5.1. TLR2

The *TLR2* gene is located on the long arm of chromosome 4 (4q31.3), coding for a protein of 784 amino acid residues of 89.9 kDa molecular weight [104].

Among the various SNPs that have been reported in *TLR2*, one is the most studied and reported in CRC susceptibility—*TLR2* Arg753Gln (+2258G/A, rs5743708) SNP [22, 49]. *TLR2* rs5743708 SNP occurs in the TIR domain of the TLR2 and has been found to result in the impaired NF-*κ*B signaling and decreased secretion of cytokines in response to the PAMPs [89].

Recently, another TLR2 genetic variation, a 22 bp deletion at position -196 to -174 of the promoter region, has been also reported in CRC (Delta22). This GV has been reported to cause the reduction in the transcription resulting in the reduced expression of the receptors in the cell [5, 105]. Zhu et al. [105] had reported that a significant association was found between the *TLR2* −196 to −174 del and cancer risk; however, it was restricted to only a subgroup of Caucasian and south Asians and not among east Asians, while Proenca et al. Proenca et al. [5] reported that *TLR2* -196 to -174 del polymorphism results in the increase in the TLR2 mRNA expression which is associated with a higher risk of developing CRC. In contrast, Pimentel-Nunes et al. [27] reported that *TLR2* (+597T/C, rs3804099) resulted in a fivefold decreased risk of CRC. Furthermore, Boraska et al. [106] found the unique microsatellite GT repeat polymorphism in the intronic region of the TLR2 gene to be associated with the CRC in the Croatian population. This repeat genetic variation is in the intron 2, 100 bp upstream of the translation start site, where GT repeats vary from 12 to 28 [107]. Based on the repeat structure, three different alleles have been categorized: (GT)16 or less as S, (GT)17-22 as M, and (GT)23 or more as L allele, and are present in different populations in different frequencies [107].

### 5.2. TLR3

The *TLR3* gene is located on the long arm of chromosome 4 (4q35.1), coding for a protein of 904 amino acid residues of 103.8 kDa molecular weight [108].

About 10 SNPs have been reported to span an entire coding region of the TLR3 gene, and among these five SNPs, rs5743305, rs11721827, rs3775290, rs3775291, and rs3775292 have been extensively studied and reported to modulate the risk of cancers including CRC [17, 109]. Wang et al. [109] in their meta-analysis reported the association of only two TLR3 polymorphisms, rs11721827 and rs3775292, with the risk of CRC. Additionally, TLR3 rs3775291 was reported to be associated with CRC-specific survival, with patients having TT genotype having a 93% increased risk of death in comparison to CC ones [110].

### 5.3. TLR4

The *TLR4* gene is located on the long arm of chromosome 9 (9q33.1), coding for a protein of 839 amino acid residues of 95.6 kDa molecular weight [111].

Of all the present SNPs in the *TLR4* gene, two nonsynonymous SNPs (+896A/G, rs4986790 and +1196C/T, rs4986791) located within the exon 3 of a gene are known to induce substitution of amino acids Asp299Gly and Thr399Ile, respectively [97, 112, 113]. The substitution of Asp299Gly disrupts the normal structure of the extracellular region of the TLR4, which may cause decreased ligand recognition or protein interaction and decreased responsiveness to lipopolysaccharide (LPS) [5, 114]. This change affects the cell surface expression of the TLR4 protein and is reported to cause inflated inflammatory response leading to severe tissue destruction [105].

*TLR4* Asp299Gly and Thr399Ile are known to cosegregate in 10% of the Caucasian populations and are reported to have positive correlation with susceptibility to several diseases [22, 49]; but neither SNP is present in Asian populations [21, 112]. Numerous studies and meta-analyses have linked TLR4 SNPs with the increased risk of CRC [22, 86, 113, 115]. In a recent meta-analysis, Sheng et al. [113] demonstrated that GG genotype of *TLR4* rs4986790 and TT genotype of *TLR*4 rs4986791 SNPs are correlated with the risk of CRC. A previous meta-analysis by Zhu et al. [105] had established a significant association of *TLR4* SNPs with cancer especially in Caucasian and south Asian populations. In addition, a recent study by Moaaz et al. [115] reported that both these SNPs are associated with the risk of CRC in the Egyptian population. In addition, a recent study in the Danish population did implicate the TLR4 and TLR2 and their interaction with the meat-rich diet with the CRC risk [116].

### 5.4. TLR5

The *TLR5* gene is located on the long arm of chromosome 1 (1q41), coding for a protein of 858 amino acid residues of 97.8 kDa molecular weight [117]. Of the many studied genetic polymorphism in the *TLR5* gene, two SNPs of the *TLR5* gene are the most important ones which have been linked with gastrointestinal cancers including CRC, which are *TLR5* rs2072493 and rs5744174 [19, 22, 89].

The rs5744174 SNP leads to the substitution of leucine to phenylalanine at 616 amino acid residues of TLR5, thereby affecting the ligand recognition capacity of the receptor, contributing to the increased inflammation and hence increase the risk of cancers [19, 28]. Since TLR5 holds a key to identify among all TLRs in being the unique receptor for bacterial flagellin protein recognition, the change in ligand binding does affect the downstream signaling and eventual secretion of IL-1*β* and IL-6. Both are key inflammatory molecules playing a direct role in inflammatory pathways of CRC carcinogenesis [28, 89]. Recently, Klimosch et al. [118] reported that these two *TLR5* SNPs did disparately affect the risk of CRC with rs2072493 being associated with worse survival while rs5744174 with better survival.

### 5.5. TLR9

The *TLR9* gene is located on the short arm of chromosome 3 (3p21.2), coding for a protein of 1032 amino acid residues of 115.8 kDa molecular weight [119]. In the TLR4 gene, two polymorphisms have been identified to play a role in the CRC tumorigenesis risk [19, 22, 89]. TLR9 is a unique receptor for the unmethylated DNA derivatives from the viruses and bacteria [24, 92]. Although the increased expression of TLR9 has been reported in the advanced CRC [120, 121], its two SNPs—*TLR9* rs187084 and rs352140—have lately been found to be associated with the inflammatory diseases which are regarded as the risk factor for CRC as well [19, 28].

## 6. Toll-Like Receptors and Therapeutics

CRC is one of the malignancies in which inflammation is considered as the pivotal risk factor [10, 23], coupled with the key role TLRs play in the inflammation-driven carcinogenesis [5, 6, 15, 16, 29, 53, 95]; targeting various TLRs with the various agonists, antagonist, and vaccine adjuvants is being currently explored as one of the strategies of cancer therapeutics [23, 122, 123].

Recently, it has been established that TLR agonists do exhibit varied therapeutic benefits as antitumor agents via their ability to activate immune cells within the tumor microenvironment on the one hand and increase the expression of cytokines on the other. The dual effects help in the activation of antitumorigenic dendritic cells, cytotoxic T lymphocytes, and natural killer cells which play an active role in the suppression of oncogenic signaling pathways ([51, 74, 75, 122]; ). Usually, there are two chief strategies which are utilized in cancer therapeutics in achieving the TLR inhibition as follows: (a) preventing the ligand binding to the TLR receptor and (b) impeding the function of various proteins located downstream of the TLR signaling [23, 51, 122, 124, 125].

The role of TLR signaling in the colorectal carcinogenesis is controversial and still limited to only few TLRs—TLR2, 3, 4, 5, and 9 [17, 27, 86, 98, 120, 126–128]. A recent advance in therapeutics has exploited both agonists and antagonists in a bid to develop an effective and long-lasting therapy for CRC [23, 51, 125].  Table 4 summarizes the different compounds which are being currently tested for the CRC therapy. While TLR agonists are used for their ability to activate the innate and adaptive immune responses, TLR antagonists are utilized for their immunosuppressive ability [86, 125, 129–131].

## 7. Conclusion

As inflammation is considered as the important risk factor for the CRC and *TLRs* playing a central role in inflammation-driven immune responses, TLRs have a universal role in carcinogenesis mechanism. Genetic polymorphisms in the important *TLR* genes especially TLR2 and TLR4 do have an effective modulatory bearing on the susceptibility to CRC, which is important in deducing them as one of the biomarkers for this malignancy.

Additionally, with the advent of new therapeutic strategies which target TLRs as legitimate targets of cancer therapy, the involvement of *TLR* SNPs and their risk modulation becomes ever more important to study and document. Numerous TLRs 2, 3, 4, 7, 8, and 9 have been identified as molecules of interest for the development of agonist, antagonist, and vaccines to develop a treatment strategy to control and prevent the development of CRC, which has a heavy bearing upon the *TLR* gene SNPs and the effects they have on immune modulation.

As this review was primarily focused on unraveling the importance of the TLR signaling and the SNPs thereof in CRC, the review is intentionally truncated with respect to the other aspects of TLR signaling. Additionally, we have discussed only those TLRs whose SNPs have been reported to be involved in CRC susceptibility. With the advancement of research on TLRs and the consequent unraveling of the role TLR SNPs play in CRC carcinogenesis, the prospects of the therapy targeting specific TLRs look promising. Currently, it would be safe to mention that combination therapy approach involving traditional drugs with the TLR agonists/antagonist may surely alleviate the benefits of treatment for CRC.

## Figures and Tables

**Table 1:** Characteristics of different human TLRs.

TLR member	TLR expression	TLR coreceptor	Gene & its location	Origin	TLR active form	Signaling adaptors
TLR1	Cell membrane	TLR2	*TLR1-6-10* *4p14*	Nonviral	TLR1-TLR2	BCAP, TIRAP, MyD88, SCIMP
TLR2	Cell membrane	TLR1, 2, 6 & 10 CD14, CD36, integrin, RP105, MBL, LBP	*TLR2* *4q31.3*	Nonviral	TLR1-TLR2 TLR2-TLR2 TLR2-TLR6 TLR2-TLR10	BCAP, TIRAP, MyD88, SCIMP
TLR3	Endoplasmic reticulum, lysosomal membrane	CD14, Mex3B	*TLR3* *4q35.1*	Viral	TLR3-TLR3	SARM, SCIMP, TRIF, TICAM1
TLR4	Cell membrane, endoplasmic reticulum, lysosomal membrane	MD2, LY96, CD14, CD36, LBP, RP105	*TLR4* *9q33.1*	Nonviral	TLR4/MD2-TLR4/MD2 TLR4-TLR6	BCAP, TIRAP, MyD88, SARM, SCIMP, TICAM1, TICAM2
TLR5	Cell membrane		*TLR5* *1q41*	Nonviral	TLR5-TLR5	MyD88, TICAM1
TLR6	Cell membrane	TLR2, CD36, LBP	*TLR1-6-10* *4p14*	Nonviral	TLR2-TLR6 TLR4-TLR6	BCAP, TIRAP, MyD88, SCIMP
TLR7	Cell membrane, endoplasmic reticulum, lysosomal membrane	CD14	*TLR7* *Xp22.2*	Viral	TLR7-TLR7	MyD88
TLR8	Endoplasmic reticulum, lysosomal membrane		*TLR8* *Xp22.2*	Viral	TLR8-TLR8	MyD88
TLR9	Endoplasmic reticulum, lysosomal membrane	CD14	*TLR9* *3p21.2*	Viral	TLR9-TLR9	TIRAP, MyD88, SCIMP
TLR10	Cell membrane, endoplasmic reticulum, lysosomal membrane		*TLR1-6-10* *4p14*	Nonviral	TLR1-TLR10 TLR2-TLR10 TLR10-TLR10	MyD88

References: Akira et al.,[29, 53]; West et al.[20]; Takeuchi and Akira, [18]; Chang, [52]; Takeda K, Akira, [31]; Gay and Gangloff, [63]; El-Zayat et al., [35, 46].

**Table 2:** TLR recognition of microbial components.

Origin	Exogenous					Endogenous
Pathogen	Bacteria	Fungus	Parasites	Viruses	Synthetic compounds	Host
Derivatives	LPS Atypical LPS Diacyl lipopeptides Triacyl lipopeptides Lipoteichoic acid Peptidoglycan Glycolipids Porins Lipoarabinomannan Flagellin CpG-DNA	Zymosan Taxol Phospholipomannan Mannan Glucuronoxylomannan	tGPI-mutin Glycoinositolphospholipids Hemozoin Profilin-like molecule	DNA dsRNA ssRNA Envelope proteins Hemagglutinin protein	Bropirimine Guanosine analogs Imidazoquinolines Loxoribine	Heat-shock protein 60, 70 Fibrinogen Hyaluronan Hemagglutinin Chromatin-IgG complexes mtDNA

TLR recognition	**TLR2, 4, 5, 6, 9**	**TLR2, 4, 6**	**TLR2, 4, 9, 11**	**TLR2, 3, 4, 9**	**TLR7**	**TLR 4**

References: Akira et al., [29, 53]; West et al., [20]; Takeuchi and Akira, [18]; Chang, [52]; Takeda K, Akira, [31]; Gay and Gangloff, [63]; El-Zayat et al., [35, 46].

**Table 3:** Summary of various TLR SNPs reported in colorectal cancer susceptibility.

Gene	Location	SNP	Major/minor allele
*TLR2*	4q32	**Delta22**	**Ins/del**
		**GT repeats**	**GT**
		rs1898830	A/G
		rs4696483	C/T
		**rs3804099**	**T/C**
		rs7656411	T/G
		rs5743704	C/A
		**rs5743708**	**G/A**

*TLR3*	4q35	**rs5743305**	**T/A**
		**rs11721827**	**A/C**
		rs3775290	A/G
		rs3775291	G/A
		**rs3775292**	**C/G**

*TLR4*	9q32-q33	rs10759932	T/C
		rs1927911	C/T
		rs5030728	G/A
		rs11536898	C/A
		rs12377632	T/C
		rs11536889	G/C
		rs1554973	T/C
		**rs4986790**	**A/G** or **A/T**
		**rs4986791**	**C/T**

*TLR5*	1q41	rs5743836	A/G
		**Rs5744168**	**G/A**
		**rs5744174**	**A/G**
		**rs2072493**	**T/A** or **T/C**

*TLR9*	3p21.2	**rs187084**	**A/G** or **A/T**
		**rs352140**	**C/A** or **C/G** or **C/T**

References: El-Omar et al., [21]; Chang, [52]; Trejo-de la et al., [19]; Skevaki et al., [94]; Mukherjee et al., [89].

**Table 4:** Summary of therapeutics targeting TLRs in CRC.

Compound	Target TLRs	Drug	Clinical phase
BCG	TLR2/4	Synthetic ssRNA	Phase I
MPL	TLR4	Synthetic ssRNA	Phase I
CBLB502	TLR5	Flagellin	Phase I
Imiquimod	TLR7	Small molecule ssRNA	Phase I/II/III
IMO2055	TLR9	CpG oligonucleotide	Phase I/II
MGN1703	TLR9	dSLIM	Phase II

References: Hennessy et al.,[130]; Gambara et al.,[129]; Vacchelli et al., [131]; Kaczanowska et al.,[124]; Moossavi et al., [125]; Li et al., [23]; Pradere et al., [74]; Gao et al., [51]; Braunstein et al.,[122].

## Data Availability

Data availability is not applicable, as it is a literature review.

## Abbreviations

ALRs: AIM2-like receptors
AP1: Activator protein 1
BCAP: B-cell adaptor for phosphoinositide
CLRs: C-type lectin receptors
CTD: C-terminal domain
DAMPs: Damage/danger-associated molecular patterns
DKs: Dendritic cells
IFN: Interferon
IKK: I*κ*B kinase
IRAK: IL-1R-associated kinase
IRF-3: Interferon regulatory factor-3
LPS: Lipopolysaccharide
LRR: Leucine-rich repeat
MAMPs: Microbe-associated molecular patterns
MAPK: Mitogen-activated protein kinases
MyD88: Myeloid differentiation primary response protein 88
NEMO: NF-*κ*B essential modulator
NF-*κ*B: Nuclear factor kappa B
NKs: Natural killer
NLRs: NOD-like receptors
NOD: Nucleotide-binding oligomerization domain
NTD: N-terminal domain
PAMPs: Pathogen-associated molecular patterns
PRRs: Pathogen recognition receptors
RACK1: Receptor for activated C kinase 1
RIP1: Receptor-interacting protein1
RLRs: Retinoic acid-inducible gene- (RIG-) I-like receptors
SARM: Sterile *α*- and armadillo-motif-containing protein
TAB: TAK1-binding protein 1
TAK1: TGF-*β*-activated kinase 1
TANK: TRAF family-member-associated NF-*κ*B activator
TBK1: TANK-binding kinase 1
TIR: Toll-interleukin-1 receptor domain
TIRAP: TIR domain-containing adapter protein
TLRs: Toll-like receptors
TRAF: TNFR-associated factor
TRAILR: Tumor-necrosis factor-related apoptosis-inducing ligand receptor
TRAM: TRIF-related adaptor molecules
TRIF: TIR-domain-containing adaptor protein that induces IFN-*γ*
TRIMs: Tripartite motif-containing proteins
TRIP: TRAF-interacting protein
XAMPs: Xenobiotic-associated molecular patterns.
